# Integrative and Multidisciplinary Care for People Living With Human T-Cell Lymphotropic Virus in Bahia, Brazil: 20 Years of Experience

**DOI:** 10.3389/fmed.2022.884127

**Published:** 2022-06-07

**Authors:** Bernardo Galvão-Castro, Maria Fernanda Rios Grassi, Ana Verena Galvão-Castro, Aidê Nunes, Ana Karina Galvão – Barroso, Thessika Hialla Almeida Araújo, Regina Helena Rathsam-Pinheiro, Ceuci Lima Xavier Nunes, Adriele Ribeiro, Monique Lírio, Noilson Lázaro Gonçalves, Sônia Lúcia Rangel, Cristiane Maria Carvalho Costa Dias, Daniele Piai Ozores, Selena Márcia Dubois-Mendes, Isabela Lima, André Luís Pereira Silva, Washington Luiz Abreu de Jesus, Fred Luciano Neves Santos, José Guilherme Reis de Oliveira, Yscela Vanessa Pimentel de Moraes, Adijeane Oliveira de Jesus, Francisco Daltro, Ney Boa-Sorte, Humberto Castro-Lima, Maria Luísa Carvalho Soliani

**Affiliations:** ^1^Escola Bahiana de Medicina e Saúde Pública, Salvador, Brazil; ^2^Laboratório Avançado de Saúde Pública, Instituto Gonçalo Moniz, Fundação Oswaldo Cruz, Salvador, Brazil; ^3^Instituto Brasileiro de Oftalmologia e Prevenção da Cegueira, Salvador, Brazil; ^4^Instituto Couto Maia, Secretaria da Saúde do Estado da Bahia, Salvador, Brazil; ^5^Associação HTLVIDA, Salvador, Brazil

**Keywords:** biopsychosocial healthcare, multidisciplinary care, HTLV, Bahia, Brazil

## Abstract

Brazil is home to the highest absolute number of human T-cell lymphotropic virus type-1 (HTLV-1)-infected individuals worldwide; the city of Salvador, Bahia, has the highest prevalence of HTLV-1 infection in Brazil. Due to the complex nature of several diseases associated with this retrovirus, a multidisciplinary health care approach is necessary to care for people living with HTLV-1. The Bahia School of Medicine and Public Health’s Integrative Multidisciplinary HTLV Center (CHTLV) has been providing support to people living with HTLV and their families since 2002, striving to ensure physical and mental well-being by addressing biopsychosocial aspects, providing clinical care and follow-up, including to pregnant/postpartum women, as well as comprehensive laboratory diagnostics, psychological therapy, and counseling to family members. To date, CHTLV has served a total of 2,169 HTLV-infected patients. The average patient age is 49.8 (SD 15.9) years, 70.3% are female, most are considered low-income and have low levels of education. The majority (98.9%) are HTLV-1 cases, and approximately 10% have been diagnosed with tropical spastic paraparesis/HTLV-1-associated myelopathy (TSP/HAM), while 2.2% have infective dermatitis and 1.1% have adult T-cell lymphoma. In all, 178 pregnant/postpartum women [mean age: 32.7 (±6.5) years] have received care at CHTLV. Regarding vertical transmission, 53% of breastfed infants screened for HTLV tested positive in their second year of life, nearly 18 times the rate found in non-breastfed infants. This article documents 20 years of experience in implementing an integrative and multidisciplinary care center for people living with HTLV in Bahia, Brazil. Still, significant challenges remain regarding infection control, and HTLV-infected individuals continue to struggle with the obtainment of equitable and efficient healthcare.

## Introduction

### Human T-Cell Lymphotropic Virus Epidemiological Status in Bahia, Brazil

Human retroviruses were identified about four decades ago. Human T-cell lymphotropic virus type-1 (HTLV-1), identified four years before the successful isolation of human immunodeficiency virus (HIV), was the first human retrovirus associated with the clinical development of disease (1, 2). HTLV-2 was isolated in 1982 and is rarely associated with disease manifestations (3). With approximately 10 million people infected worldwide, despite the advances made in the scientific understanding of this viral infection, HTLV-1 and its associated diseases remain extremely neglected (4, 5). Most geographic regions affected by this virus, except Japan, are classified as middle- or low-income, and infected persons possess low levels of education and income (6). The geographic regions in which HTLV-1 is primarily endemic are Japan, the Caribbean, South and Central America, Equatorial Africa, the Middle East, Melanesia, and Australia. Brazil is considered the country with the highest absolute number of people (approximately one million) living with HTLV-1 (PLHTLV) (7, 8). The first Brazilian cases of HTLV-1 infection were detected in 1986 in the state of Mato Grosso do Sul (9). Several studies conducted by Brazilian researchers have reported that infection is prevalent throughout the national territory, with higher prevalence noted in the north and northeast regions (10, 11). In Bahia, there is strong evidence supporting multiple post-Columbian introductions of HTLV-1 during the slave trade between the 16th and 19th centuries (12–15). This state also has the highest prevalence of HTLV-1, with nearly 130,000 PLHTLV (16). In the city of Salvador, the state capital, a general population study estimated 40,000 HTLV-1-infected individuals, corresponding to a prevalence of 1.7% (13). This study also found that prevalence increases with age, reaching 8.4% in those aged over 51 years, and noted a higher prevalence in individuals with lower income, less education, and poorer living conditions (13). Sexual transmission appears to be the predominant route of HTLV-1 infection in Salvador (17).

Efforts by Brazilian scientists have contributed to a better understanding of the epidemiology, clinical and laboratory aspects as well as pathogenesis of this infection, thereby raising awareness of HTLV-1 as a serious public health problem in Brazil (18). Although screening for HTLV-1 at blood banks became mandatory in Brazil since 1993 (19), only recently has the WHO/PAHO considered HTLV-1 infection to be a serious health problem, prompting the Brazilian Ministry of Health to implement additional measures to control infection (20, 21).

It is important to note that while there is no cure or effective vaccine for HTLV-1 infection, pharmacological and non-pharmacological treatments help minimize patient suffering and improve the quality of life (QoL) of this neglected population. Furthermore, while additional public health measures are required to prevent and/or control the spread of HTLV-1 infection, health care provisions for patients also remain a challenge (22, 23).

### Why Do People Living With Human T-Cell Lymphotropic Virus Need Integrative Healthcare?

Human T-cell lymphotropic virus type-1 infection can cause proliferative disease, such as adult T-cell leukemia, and inflammatory disorders, including HTLV-1-associated myelopathy/tropical spastic paraparesis (HAM/TSP), uveitis, and infectious dermatitis (24–28). Other diseases, such as polymyositis, sinusitis, keratoconjunctivitis sicca (KCS) and a variety of pulmonary disorders, including bronchiectasis, have also been associated with HTLV-1 (8, 29, 30). The myelopathy associated with HTLV-1 infection leads to a series of systemic alterations, including urologic changes (nocturia, frequency, urgency, and urinary incontinence), constipation and sexual dysfunction (31–33), as well as pain, muscle spasticity, postural changes and reduced muscle strength, functional mobility and flexibility (34–36). Moreover, it has been reported that HTLV-1-infected individuals may present some degree of immunosuppression, since several infectious diseases, such as tuberculosis, strongyloidiasis and scabies occur more frequently or are more severe in affected individuals (5, 8, 37, 38). In addition, HTLV-1 infection predominantly affects older individuals for whom comorbidities such as diabetes, systemic arterial hypertension and overweight are frequent (39).

People living with human T-cell lymphotropic virus may also present a higher prevalence of psychiatric/psychological disorders than the general population, including signs of psychological distress, anxiety, sleep and psychosomatic disorders, suicide ideation, low self-esteem, and depression (40–43). Indeed, the prevalence of major depression in HTLV-1 patients seen at CHTLV and in the general population of Salvador was estimated at around 30 and 12%, respectively (44). In addition, it is important to note that the vertical transmission of HTLV-1 is known to provoke biopsychosocial disorders in parents and children that require long-term multidisciplinary care (20, 45, 46). All of these conditions can negatively affect patients’ QoL and consequently lead to alterations in the performance of daily activities, sleep, and self-perception of health (41, 45, 47). In conclusion, the complex nature of HTLV infection requires an integrative and multidisciplinary approach to the biopsychosocial care of PLHTLV.

## The Creation of CHTLV

The Bahiana School of Medicine and Public Health (EBMSP), created in 1952, is a private, non-profit higher education institution dedicated to teaching, research and providing health-based extension programs. The EBMSP curriculum consists of seven undergraduate courses (Biomedicine, Dentistry, Medicine, Nursing, Physiotherapy, Psychology, and Physical Education). EBMSP also develops scientific, cultural, and socio-environmental activities that encourage societal and community interaction by working with diverse sectors and professions with the ultimate goals of contributing to social change and promoting health (48).

In 2002, as part of its social mission in collaboration with the Gonçalo Moniz Institute of the Oswaldo Cruz Foundation (IGM-Fiocruz), EBMSP established the Integrative Multidisciplinary HTLV Center (CHTLV) to provide an array of health services for PLHTLV and their families. CHTLV was inspired by the Interdisciplinary HTLV Research Group (GIPH) of Belo Horizonte (state of Minas Gerais), which initiated activities in January 1997 (49). The center provides patients with comprehensive biopsychosocial care under the guidance and support of the Brazilian Unified Health System (SUS) (50). The country’s social welfare system, guaranteed by the 1988 Federal Constitution, encompasses SUS together with social security and social assistance programs. Social assistance and public health are funded by the federal government, while social security is funded through taxpayer contributions (51). While a range of social benefits (disability retirement and income tax exemptions) are provided for people living with HIV, due to the invisible and neglected status of HTLV infection, HTLV receive no specific social welfare benefits (52).

## Multidisciplinary Approach to Caring for People Living With Human T-Cell Lymphotropic Virus

Considering that the general guidelines providing for the clinical follow-up of HTLV patients have been well established by the Department of STD, AIDS and Viral Hepatitis (21), here we focus on presenting our experience regarding integrative and multidisciplinary care, highlighting the role of psychologists, nurses, physical therapists, and social workers, as well as services for pregnant women.

**FIGURE 1:**
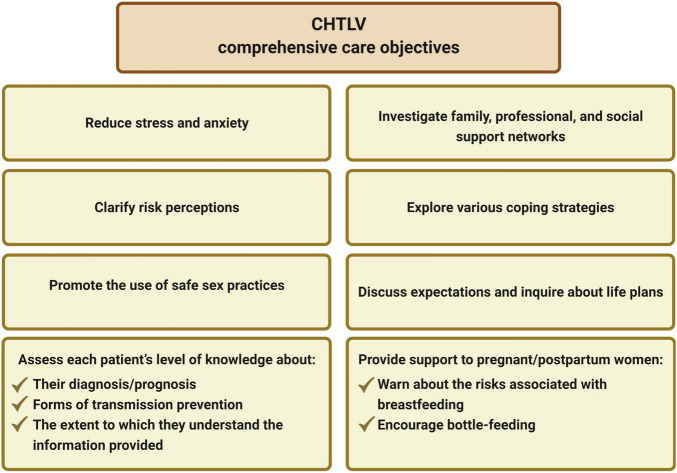
Goals of comprehensive care at the Integrative Multidisciplinary HTLV Center (CHTLV).

The Singular Therapeutic Project is a set of proposals detailing articulated therapeutic approaches for individuals, families or groups, arising from collective discussions among members of an interdisciplinary team (53). In accordance with this approach, all members of the CHTLV multidisciplinary/interdisciplinary team work together to develop an individualized care plan that prioritizes each patient’s needs. Follow-up appointments are scheduled to monitor patients’ health status and QoL, as well as to assess disease progression. The ultimate goals of CHTLV’s comprehensive care are highlighted in Figure 1. Most patients seen at CHTLV are referred by blood banks and other primary care facilities. They are initially seen by a psychologist who answers patients’ questions, provides general information about HTLV as well as serologic counseling both before and after diagnosis, and educates individuals about infection prevention measures.

**FIGURE 2:**
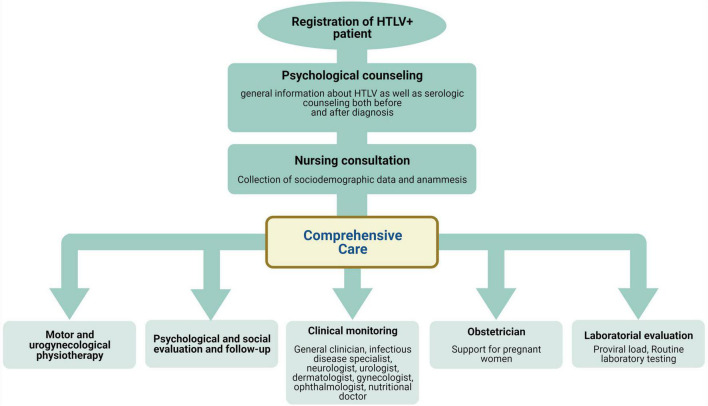
Flowchart detailing biopsychosocial comprehensive care for HTLV-1-infected individuals followed at Integrative Multidisciplinary HTLV Center (CHTLV).

In accordance with the nursing process, patient sociodemographic data and current and past health history are obtained during the nursing consultation (53). Nurses also aim to promote self-help activities, support recovery from HTLV-related diseases, and help patients adapt to the effects of infection. Psychologists provide individual and group psychotherapy for patients suffering from mood fluctuations and emotional instability. Patients with depression and other psychiatric disorders are referred to a psychiatrist who provides further treatment in conjunction with psychologists. Nurses, psychologists and psychotherapists co-host monthly self-care workshops that directly engage patients in the pursuit of promoting better health and address daily care issues, such as fall prevention, healthy eating, physical exercise, and coping with stress. Physical therapists together with nurses and neurologists assess functional capacity by analyzing patients’ gait and mobility using the Kurtzke and Osame scales (54, 55) as well as specialized questionnaires to evaluate urinary and/or bowel changes. Patients undergo routine laboratory testing at EBMSP facilities, including HTLV-1 proviral load quantification (performed in collaboration with Fiocruz-Bahia). Complementary exams, such as cerebrospinal fluid analysis, are performed when indicated at SUS-affiliated clinics. Patients are evaluated at CHTLV by a multidisciplinary team of specialists in nursing, psychology, physical therapy, infectious diseases, neurology, ophthalmology, dermatology, urology, gynecology, obstetrics, nutrition, stomatherapy, and social work (Figure 2).

Since most individuals who receive care at CHTLV are unaware of the eligibility to receive benefits from the Brazilian Welfare System due to HTLV-related disabilities, social workers play an important role in counseling PLHTLV on the obtainment of such benefits.

## Profile of People Living With Human T-Cell Lymphotropic Virus Seen at the CHTLV

Since its inauguration in 2002, a total of 2,169 HTLV-infected patients have been seen at CHTLV, approximately less than 50% of whom are evaluated regularly. Of these, 2,145 (98.9%) are cases of HTLV-1 infection, while 24 (1.1%) individuals were diagnosed with HTLV-2. Almost all patients (98%) reside in the city of Salvador (Bahia, Brazil), are aged between 5 and 93 years [mean: 49.8 (SD 15.9)], and 70.3% (1,525) are female. Most (84.6%) self-reported black or brown skin color, 73% had less than 8 years of schooling, and half earned the equivalent of one Brazilian monthly wage (∼US$200). Thus, most are considered low-income and possess low education levels. The PLHTLV seen at CHTLV face significant difficulties to attend consultations due to physical limitations and/or lack of access to public transportation.

The clinical profile observed in CHTLV patients is similar to that of another center in Salvador (31, 56, 57): ∼10% have TSP/HAM, 2.2% have infective dermatitis, and 1.1% have adult T-cell lymphoma. The annual incidence density of TSP/HAM was found to be 6.9 per 1,000 PLHTLV, similar to that reported in Minas Gerais (58, 59). Several bladder voiding disorders, such as urinary urgency (78%), nocturia (73.8%), urge incontinence (70.7%), the sensation of incomplete bladder emptying (65.2%) and pollakiuria (59.1%), detrusor hyperactivity (69.4%), significant delay after bladder emptying (52.9%), hyposensitivity (45.9%), and detrusor sphincter dyssynergia (39.3%), were observed in a representative sample of PLHTLV; all these disorders were found to occur significantly more frequently in patients with TSP/HAM (60). Moreover, urge incontinence in women was shown to negatively impact several aspects of QoL, including general health perception, performance of daily activities, sleep and disposition, emotional state, and social relationships (61). Common HTLV-associated dermatologic changes include xerosis (23.4%), seborrheic dermatitis (19%), dermatophytosis (13%), scabies (7.6%), and pityriasis versicolor (7.1%) (62). The overall prevalence of KCS was reported to be 31.7%, with higher rates observed in TSP/HAM patients even after adjusting for age, sex, time of HTLV-1 diagnosis and schooling. Proviral load, low corrected visual acuity, burning and/or eye pain and itching were all significantly more frequent in patients with KCS (29).

Based on the high frequency of KCS and the difficulty in diagnosing dry eye, an algorithm, using low-cost and minimally invasive tools, for the diagnosis of this disease was developed. Briefly, a sequence of tests in three stages was proposed rather than performing all tests simultaneously. First, patients are submitted to the Ocular Surface Disease Index (OSDI) questionnaire and the tear breakup time test (TBUT). If results from both tests are normal, a diagnosis of KCS can be excluded. However, if the OSDI and/or TBUT results are abnormal, a Schirmer I test is performed, with positivity confirming KCS diagnosis. Lastly, in patients with an indeterminate diagnosis (i.e., negative Schirmer I test), Rose Bengal staining must then be performed (63). Unexpectedly, uveitis was diagnosed in only 2.8% of the patients seen at CHTLV (64). However, a more accurate diagnostic approach yielded an increased prevalence of 7.0% in the patients seen at CHTLV (65). A 2012 study identified that PLHTLV commonly present poor oral health; in addition to dry mouth and decreased salivary flow, other manifestations, such as periodontal disease, gingival attachment loss, and tooth mobility are frequent findings. Moreover, a direct relationship between proviral load in saliva and oral manifestations has been observed (66).

With respect to HTLV-1 transmission, a pilot project at CHTLV identified a prevalence of 53.5% (146/273) for family aggregation of HTLV-1 infection. Greater TSP/HAM prevalence was observed among index cases. Probable sexual and vertical transmission was estimated at 44.4 and 22.3%, respectively. Index and family cases reported a history of 1–5 partners, with no condom use prior to the diagnosis of HTLV-1. However, enhanced adherence to the use of condoms was described after HTLV-1 diagnosis (67). A study investigating risk for HTLV-1 infection in females suggested that more than three lifetime sexual partners, age ≤18 years at time of first sexual intercourse and engaging in anal intercourse were relevant risk factors (68). In addition, the prevalence of human papillomavirus (HPV) infection was higher in HTLV-1-infected women (69).

Human T-cell lymphotropic virus type-1-infected patients seen at CHTLV were estimated to face an overall 2.6 times greater relative risk of developing tuberculosis (38). The prevalence of strongyloidiasis in PLHTLV was found to be much lower than that previously reported in other Brazilian cities, which is probably due to improvements in sanitation systems (70).

Studies investigating depression in our cohort identified a prevalence ranging from 34.1 to 38.0% (42, 43). While no associations between the presence of TSP/HAM and a diagnosis of depression were observed in a global analysis of PLHTLV, a stratified analysis revealed a greater prevalence of depression among individuals with HAM/TSP aged between 18–39 years (PR: 2.59; CI 95%: 1.36–4.95) (43). Pain, a common finding in infected individuals, was reported by 84.3% of PLHTLV (71). Pain and postural changes affect many aspects of QoL and negatively impact an individual’s ability to walk and work (72). Nevertheless, a randomized crossover clinical trial demonstrated that Pilates exercises can improve lower back pain and QoL in PLHTLV (73). In addition, it was demonstrated that home exercises oriented by a guidebook may benefit posture, functional mobility and gait parameters in people with TSP (36).

### Sexual and Vertical Transmission of Human T-Cell Lymphotropic Virus Infection in the State of Bahia, Brazil

The rate of HTLV-1 infection in pregnant women in the state of Bahia ranks among the highest in Brazil, with several evaluations reporting prevalence between 0.84 and 1.05% (15, 74–76) by contrast, HTLV-2 (0.03%) prevalence appears to be low (75).

CHTLV currently serves 178 pregnant/postpartum women. The sociodemographic characteristics of the pregnant women are similar to those of the other women seen at CHTLV, with the exception of younger age [mean (SD): 32.7 (±6.5) years]. A preliminary retrospective study conducted between 2003 and 2012 to evaluate the impact of breastfeeding duration on the vertical transmission of HTLV-1 included a representative sample of postpartum women and their 50 children (77). Of the 50 children evaluated, 33 were not breastfed; 53% of the breastfed infants were serologically reactive for HTLV at two years of follow-up, nearly 18 times the rate found in non-breastfed infants. All women who breastfed their children were not tested for HTLV during prenatal care (77).

The crucial step of establishing prenatal screening for HTLV in the state of Bahia was supported by projects carried out between 2006 and 2010 (financed by FAPESB to conduct research efforts for SUS, coordinated by CHTLV/EBMSP in collaboration with APAE Salvador (the Association of Parents and Friends of Handicapped Children of Salvador; Associação de Pais e Amigos dos Excepcionais de Salvador). Screening for HTLV *via* dried blood spot testing has been demonstrated as a secure, viable and low-cost method of increasing pregnant women’s access to serological testing during prenatal care, helping to prevent vertical transmission (78). Bahia state health secretariat (SESAB) currently recommends that HTLV-inflected pregnant women should be followed monthly at a multidisciplinary health care service until the 30th week of gestation, fortnightly between the 30th and 36th weeks and then weekly from the 36th week until delivery (79).

**FIGURE 3:**
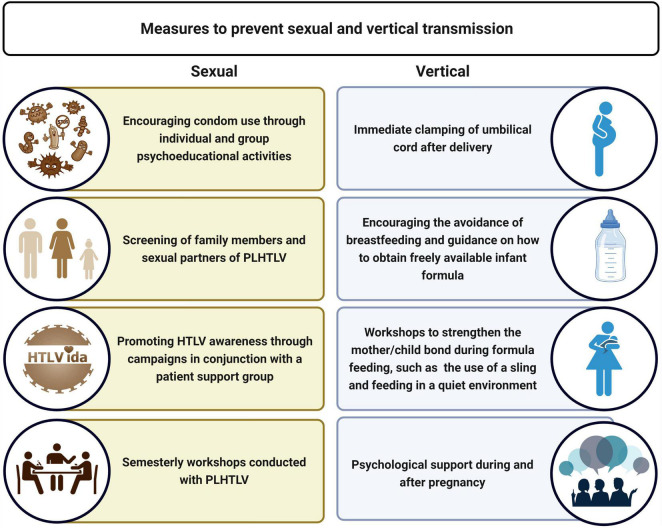
Actions conducted by the Integrated Multidisciplinary HTLV Center (CHTLV) to prevent sexual and vertical transmission. HAM/TSP, HTLV-1-associated myelopathy/tropical spastic paraparesis; KCS, keratoconjunctivitis sicca.

In April 2013, the SESAB recommended the suspension of breastfeeding by HTLV-positive pregnant women and implemented the provision of milk formula for the first year of the newborn’s life. Campaigns in Brazil commonly recommend breastfeeding as a measure to prevent malnutrition, and also portray breastfeeding as an act of maternal love. In addition to stigmatization due to social and cultural pressures, the inability to breastfeed can engender fears about maternal bonding with newborns. On the other hand, continuing to breastfeed may provoke feelings of guilt associated with the responsibility of viral transmission to children. These sensitive issues present challenges to the prevention of mother-to-child transmission. Accordingly, integrative care for HTLV-1-infected women during pregnancy and the postpartum period is crucial to infection prevention across multiple generations. Thus, great effort has been made to deconstruct longstanding beliefs to give new meaning to bottle feeding as a benevolent act of maternal love in order to preserve newborn health. Importantly, no consensus has been reached regarding the benefits of cesarean delivery in preventing the vertical transmission of HTLV. However, immediate clamping of the umbilical cord following delivery has been strongly recommended (5). In addition to significant efforts to control the vertical transmission of HTLV-1, CHTLV has also conducted several actions designed to prevent sexual transmission (Figure 3).

## CHTLV and the COVID-19 Pandemic

People living with human T-cell lymphotropic virus have not been considered as a priority group in the COVID-19 vaccination campaign in Brazil. Working together with the advocacy group HTLVida,^1^ CHTLV has strived to vaccinate as many PLHTLV as possible in accordance with the priorities established by the Brazilian Ministry of Health (older age, comorbidities, impaired ambulation, etc.). Of the 258 patients who are members of the HTLVida association, 81 were successfully vaccinated with the help of this organization and CHTLV, according to the following criteria: 37 (46%) due to older age, 27 (33%) because of impaired mobility, 8 (10%) due to comorbidities, 6 (7%) were immunosuppressed, and 3 (4%) belonged to specific groups. Of these 81 PLHTLV, we contacted 52 individuals: 41/52 (79%) have HAM/TSP and 10/52 (19%) contracted COVID-19. Vaccinations included: 14 (27%) CoronaVac, 24 (46%) AstraZeneca/Oxford, 11 (21%) Pfizer, and 2 (4%) Janssen. As of February 2022, 24 (46%) HTLV-1 carriers had received two doses, while 25 (48%) had received a booster shot. Side effects were reported by 38 (73%) patients, and the most prevalent symptoms were as follows: 26 (50%) arm pain, 11 (21%) headache, 10 (19%) fever, 9 (17%) excessive tiredness, 6 (11%) chills, and 5 (10%) muscle pain.

In accordance with recommendations issued by the Brazilian government due to the COVID-19 pandemic, EBMSP implemented a contingency plan that severely limited in-person patient care at CHTLV. However, the center implemented telehealth consultations to follow patients regularly seen at CHTLV. The remote care provided by EBMSP/CHTLV allowed for the early diagnosis of complications and stimulated adherence to treatment in PLHTLV. In addition, telehealth consultations helped alleviate some psychological distress caused by social distancing (80). Moreover, using online videoconferencing tools, it was possible to monitor patients who were already undergoing physiotherapy to treat overactive bladder. Patients were supervised during electrostimulation and instructed on how to perform pelvic floor exercises as well as bladder reeducation. As the implementation of telehealth services allowed for greater patient adhesion to follow-up, CHTLV plans to move to a hybrid format of in-person and online consultations that will facilitate the provision of care to PLHTLV who face difficulties in terms of locomotion and access to public transportation.

## Multidisciplinary Research Efforts to Minimize Suffering and Improve People Living With Human T-Cell Lymphotropic Virus Quality of Life

**FIGURE 4:**
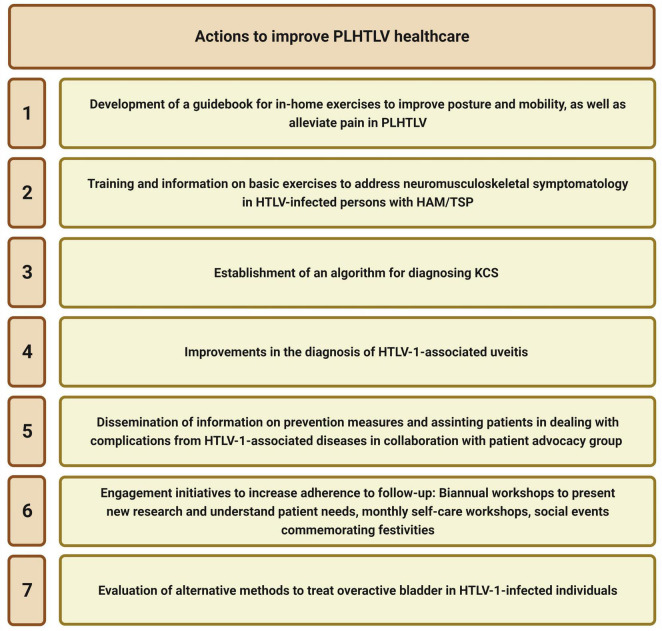
Measures taken by CHTLV to control infection and minimize PLHTLV suffering. PLHTLV, people living with human T-cell lymphotropic virus; CHTLV, Integrative Multidisciplinary HTLV Center.

The mostly multidisciplinary research carried out at CHTLV has been designed to alleviate patient suffering and improve QoL (Figure 4). The research produced in conjunction with CHTLV has contributed to a better understanding of this endemic disease, as well as aided in the training and qualification of academics and professionals working in diverse areas of health.

## Role in Supporting Professional Development

To promote professional development, some actions have been carried out by EBMSP in conjunction with CHTLV, such as: (1) the inclusion of HTLV in research lines of postgraduate courses; to date, 42 and 19 students have obtained their master’s and Ph.D. degrees respectively; (2) the provision of university-based continuing education training for graduate students in psychology, physiotherapy, nursery, and medicine; (3) weekly meetings to discuss HTLV-related cases with undergraduate and graduate students, and professionals working at CHTLV; (4) financial support for professionals and students to participate in scientific meetings, and scholarships for undergraduate students to develop scientific projects.

## Actions to Reduce Discrimination

It is known that PLHTLV suffer high rates of discrimination, mainly due to their vulnerable status as reflected by sociodemographic profile, which becomes intensified as a result of HTLV infection. The interdisciplinary group at CHTLV carries out actions that directly or indirectly seek to minimize prejudice and enable better QoL.

Psychoeducational therapeutic intervention is offered to patients and their loved ones, providing information and support to better understand and cope with not only their illness, but also social prejudice. Unfortunately, some patients attempt to hide HTLV infection from their relatives, straining family relationships.

Our psychotherapeutic group seeks to strengthen PLHTLV in their fight against prejudice, who are often socially isolated from their family, circle of friends and work colleagues. In psychotherapy, they share common experiences, discuss coping strategies and establish, both among themselves and with professionals, bonds reinforced by a qualified support network.

In addition, actions are taken to inform the general population about the virus, demystifying false ideas and encouraging preventative measures.

Another action carried out by the EBMSP nursing extension program, in partnership with the HTLVida association, promotes artistic activities and art exhibitions as a way to value autonomy and help PLHTLV cope (9a61047e-67fb-4fb5-b426-cd0bba5aa9c5.mp4).

## Collaboration With Research Centers, Laboratories, Policymakers, and Patient Representative Groups

Collaboration with other research centers has allowed us to not only train health care workers and laboratory staff and promote international exchange with students, researchers, and professionals, but also develop relevant scientific research *via* a regional, national and international network.

Members of the CHTLV multidisciplinary team have participated in several public engagement sessions with policymakers organized by the city of Salvador and the Bahia state legislative assembly to encourage the adoption of HTLV-related public health measures. Some team members who advocate for PLHTLV also participate on Bahia State Health Secretariat advisory committees.

CHTLV organizes biannual meetings with PLHTLV to discuss their needs and report on research findings, and co-sponsors commemorative events with HTLVIDA, a patient support group created in 2010 with almost 600 members. Events include Brazilian HTLV Day (23 March), the Annual Day for the Prevention and Control of HTLV in Salvador (28 September), and World HTLV Day (10 November). In addition, CHTLV/HTLVIDA also promote social events commemorating festivities, such as Christmas and St. John’s Day, which are popular holidays in northeastern Brazil.

## Challenges Faced by CHTLV in Providing Care for People Living With Human T-Cell Lymphotropic Virus

People living with human T-cell lymphotropic virus require multidisciplinary care involving multiple medical specialties and laboratory and imaging tests of moderate complexity. For instance, due to structural problems associated with the national unified health care system (SUS) in Brazil, the time required to perform exams, such as MRI or CT, which are essential for patient treatment, is prolonged. Moreover, the scarcity of professionals working in some specialized areas requires CHTLV to meet patients’ needs by collaborating with trained physicians who desire to further their academic studies. In the absence of these types of collaborations, patient services risk being disrupted.

As PLHTLV suffer from a chronic condition for which there is no specific treatment or cure, they require ongoing care—yet CHTLV is not able to provide care to patients in need (it has been estimated that 50,000 individuals have HTLV in the city of Salvador alone). High demand for services may also compromise the continuity of patient care. Improved integration among the various public health services in the state of Bahia will be necessary to ensure the provision of high-quality care to this neglected population.

## Conclusion

Brazil is a continental country with great ethnic and economic differences that lead to exacerbated health inequities. Taking into account that integrative health care involves not only individuals, but also their families and communities, including biological, psychological, and social health needs, we recognize the enormous challenge that Brazil faces in implementing efficient and humane health care for PLHTLV.

We believe that the actions implemented by CHTLV have contributed to (a) improving the quality of healthcare services provided to HTLV carriers; (b) reducing prejudice and discrimination against HTLV carriers; (c) controlling HTLV infection in vulnerable populations throughout the state of Bahia; (d) the generation and dissemination of scientific and technological knowledge about HTLV infection; (e) the implementation and development of public policies aimed at raising HTLV awareness; (f) the training/education of undergraduate/graduate students; and (g) informing public policymakers about the prevalence of infection, in line with the guiding principles of SUS.

We strongly recommend that comprehensive efforts to provide healthcare services for PLHTLV include laboratory diagnosis, counseling (including psychological and social support), clinical and oral health monitoring (for both asymptomatic and symptomatic patients), as well as pharmacological and non-pharmacological therapeutic measures to alleviate suffering and improve QoL in this extremely neglected population.

It is important to strengthen the roles played by patient advocacy groups in interacting with patient care providers to better understand patient concerns, as well as to influence the establishment of pertinent public health policies for PLHTLV.

## Data Availability Statement

The raw data supporting the conclusions of this article will be made available by the authors, without undue reservation.

## Ethics Statement

The studies involving human participants were reviewed and approved by the Comitê de Ética em Pesquisa em Seres Humanos da Bahiana. Written informed consent from the patients/participants or their legal guardian/next of kin was not required to participate in this study in accordance with the national legislation and the institutional requirements.

## Author Contributions

BG-C designed and supervised the work. BG-C and MFRG wrote the first draft. All authors contributed to the article and approved the submitted version.

## Conflict of Interest

The authors declare that the research was conducted in the absence of any commercial or financial relationships that could be construed as a potential conflict of interest.

## Publisher’s Note

All claims expressed in this article are solely those of the authors and do not necessarily represent those of their affiliated organizations, or those of the publisher, the editors and the reviewers. Any product that may be evaluated in this article, or claim that may be made by its manufacturer, is not guaranteed or endorsed by the publisher.

## Footnotes

1
http://www.htlvida.org/
